# Rotenone accelerates endogenous α-synuclein spreading and enhances neurodegeneration in an intra-striatal α-synuclein preformed fibril injected mouse model of Parkinson’s disease

**DOI:** 10.3389/fncel.2025.1624593

**Published:** 2025-10-03

**Authors:** Engila Khan, Nada Radwan, Mustafa T. Ardah, Tohru Kitada, M. Emdadul Haque

**Affiliations:** 1 Department of Biochemistry and Molecular Biology, College of Medicine and Health Sciences, United Arab Emirates University, Al Ain, United Arab Emirates; 2 Department of Medical Laboratory Sciences, Faculty of Allied Medical Sciences, Al-Ahliyya Amman University, Amman, Jordan; 3 Otawa-Kagaku, Parkinson Clinic and Research, Kamakura, Japan

**Keywords:** α-synuclein, mouse model, neurodegeneration, Parkinson’s disease, rotenone

## Abstract

Prominent histopathological features of Parkinson’s disease (PD) include the presence of Lewy bodies, intra-neural protein aggregates mainly composed of α-synuclein (α-syn), and cell death of dopaminergic neurons. Epidemiological studies have revealed a correlation between exposure to environmental neurotoxins, such as rotenone, and an increased risk of developing PD. In this study, we evaluated the role of rotenone in α-syn spreading and accumulation, with the aim of developing a mouse model of accelerated PD. Human α-synuclein pre-formed fibrils (PFF) were injected into the mouse striatum by stereotactic surgery. Rotenone (2.5 mg/kg-body-weight) was administered intraperitoneally once daily for four consecutive weeks one day or three weeks after the PFF injection. Brains were collected twenty-four hours after the last injection for immunohistochemical analysis. In this study, rotenone significantly synergized PFF induced α-syn spreading, neuroinflammation, in addition to augmented loss of dopaminergic neurons along the nigrostriatal pathway.

## 1 Introduction

The incidence of Parkinson’s disease (PD), a progressive neurodegenerative disorder, is rising at alarming rate each year (Zheng and Chen, 2022). Given PD’s complex etiology, with both genetic and environmental factors playing an essential role in the onset of the disease and its progression (Kaur et al., 2019), a number of *in vivo* and *in vitro* experimental models have been employed to decipher and improve our understanding of the underlying pathological mechanisms. A major neuropathological feature of PD is the presence of Lewy bodies, majorly comprising misfolded α-synuclein (α-syn) proteins. α-syn misfolding and aggregation had been linked to a variety of pathological consequences of PD, especially neuroinflammation and dopaminergic neurodegeneration (Forloni, 2023). Thus, the role of α-syn in the investigation of PD pathology and potential therapeutical approaches is crucial in PD research.

Epidemiological studies exploring the role of environmental factors in the progression of PD have found an important correlation between exposure to environmental toxicants and presentation of PD-like symptoms (Bastías-Candia et al., 2015; Dhillon et al., 2008; Nandipati and Litvan, 2016; Tanner et al., 2011; Wirdefeldt et al., 2011). Rotenone, a natural pesticide, mimics PD manifestation primarily via oxidative stress conferred by its inhibition of complex 1 in the electron transport chain (Sherer et al., 2003).

The utilization of intra-cranial injection of α-syn pre-formed fibrils (PFF) in PD animal modeling has gained wide popularity in PD research. The neurotoxicity of PFF is primarily attributed to the nucleation reaction of indigenous α-syn with supplemented PFF (Steiner et al., 2011; Wood et al., 1999). Additionally the retrograde transmission capacity of α-syn aggregates along the nigrostriatal pathway (Henrich et al., 2020; Schaser et al., 2020) and α-syn-mediated dysfunction and neurotoxicity offers better insights into comprehending and modulating the chronic degenerative course of the disease (Khan et al., 2023).

A successful demonstration of this technique was done by Luk et al. (2012), where intra-striatal injection of synthetic mouse α-syn PFF in C57BL6J mice induced a Lewy body (LB) like pathology and spreading of α-syn in inter-connected brain regions. However, it took around six months for the neuropathological features to become apparent, which is a moderately long time. To this end, recently, our lab developed a combinatorial PD model via intra-striatal PFF injection and intra-peritoneal (IP) administration of a low-dose of neurotoxin MPTP in C57BL6J mice (Merghani et al., 2021). It was the first such study to explore this novel method of PD induction that showed both α-syn spreading and neurodegeneration, providing a new avenue for investigating the underlying molecular mechanisms and α-syn spreading in the substantia nigra pars compacta (SNC) within a relatively short timeframe.

In current study, we examine the impact of rotenone, a naturally derived mitochondrial toxin and widely used pesticide on enhancing α-syn pathology and associated neurodegeneration in a PFF-injected mouse model. Unlike MPTP, whose toxic effects are largely confined to dopaminergic (DA) neurons, rotenone induces mitochondrial dysfunction across multiple cell types, thereby modeling a broader spectrum of PD pathology. Growing evidence supports Braak’s staging hypothesis, which proposes that α-syn pathology may begin in peripheral tissues, such as the olfactory epithelium and gastrointestinal tract, before progressing to the central nervous system via autonomic or enteric nerves (Braak et al., 2003). Rotenone’s systemic action aligns with this theory, offering a model that better reflects the multi-site, progressive nature of PD. Furthermore, unlike MPTP, a synthetic by-product of illicit drug synthesis with limited environmental relevance, rotenone has been implicated in human PD through epidemiological studies linking pesticide exposure to increased disease risk (Bastías-Candia et al., 2015; Dhillon et al., 2008; Nandipati and Litvan, 2016; Tanner et al., 2011; Wirdefeldt et al., 2011). Rotenone’s physicochemical properties, particularly its lipophilicity, enable it to cross biological barriers and induce PD-like features in animal models (Miyazaki and Asanuma, 2020).

Thus, this study aims to evaluate whether a low, sub-toxic dose of rotenone can accelerate PFF-induced α-syn spreading, accumulation, and aggregation over a short period in a mouse model. Specifically, we examine the extent to which rotenone enhances PFF-mediated α-syn pathology and its associated outcomes, including DA neurodegeneration, nigrostriatal tract damage, and neuroinflammation.

## 2 Materials and methods

### 2.1 Animals

Male C57BL/6J mice, aged 2 to 2.5 months and weighing approximately 25–30 grams at the start of the experiment, were obtained from the Animal Housing Facility at the College of Medicine and Health Sciences. Six animals were assigned to each experimental group. Mice were housed individually under standard conditions, maintained on a 12-h light/dark cycle, with ad libitum access to food and water throughout the duration of the experiment.

Ethical approval was obtained from the UAEU Animal Ethics Committee (ERA_2021_8404), and procedures were conducted in accordance with the Animal Ethics committee’s guidelines. All surgical procedures in this study were approved by the UAEU Animal Ethics Committee (ERA_2021_8404) (November 30, 2021).

### 2.2 Experimental design

**Figure 1:**
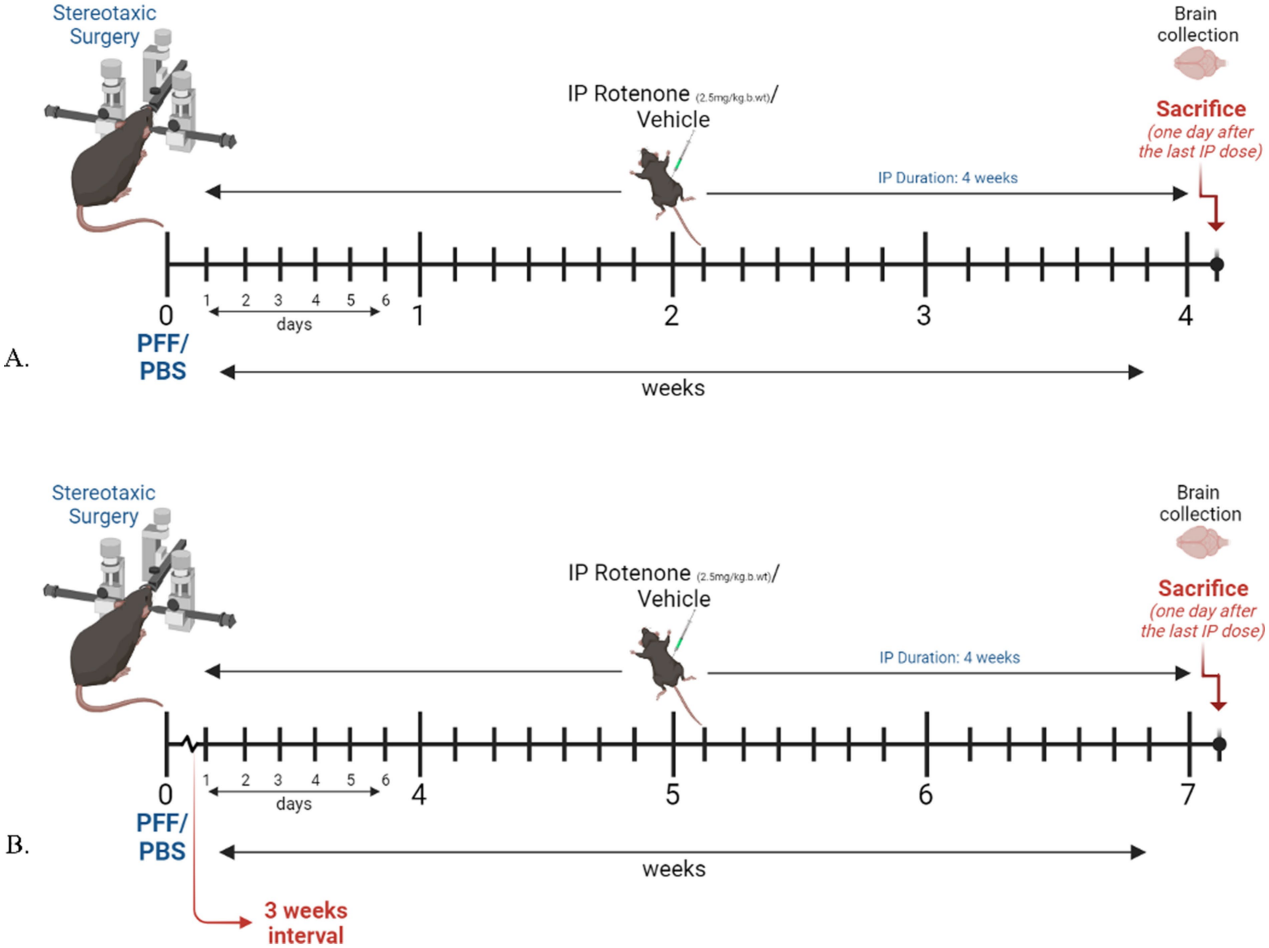
Experimental design and timeline. Figure illustrated using BioRender. **(A)** Model system 1, where rotenone administration starts one day after surgery. **(B)** Model system 2, in which rotenone administration starts 3 weeks after PFF/PBS intracerebral delivery.

Human α-syn PFF seeds (5 μg) were injected into the right dorsal striatum (STM) of C57BL6J mice by stereotaxic surgery. One day (Model system 1) or three weeks after PFF injection, (Model system 2), rotenone (2.5 mg/kg-body-weight) was administered intra-peritoneally for four consecutive weeks, once daily (Figure 1). Twenty-four hours after the final rotenone injection, animals were perfused and sacrificed for immunohistochemical (IHC) analysis.

### 2.3 Intra-peritoneal administration of rotenone

Mice were administered either rotenone (R8875-1G, Sigma Aldrich, Merck, Burlington, MA, United States) at a dose of 2.5 mg//kg-body-weight or an equivalent volume of the vehicle daily for four consecutive weeks through IP route. A rotenone stock solution [62.5 mg dissolved in 5 mL of DMSO (dimethyl sulfoxide); Sigma Aldrich, St. Louis, MO, United States] giving a concentration of 12.5 mg/mL was prepared, aliquoted into tubes (each containing 100 μL), and stored at −80 degrees. Daily, one aliquot was thawed, mixed with 4.9 mL of sunflower oil (Noor Sunflower 100% pure oil, Emirates Refining Co., Ltd., Sharjah, UAE) (1 in 50 dilutions, giving a concentration of 2.5 mg/mL), and administered to animal at a dose of 2.5 mg/kg-body-weight. For the vehicle, an equivalent amount of DMSO was mixed with oil and administered to animals.

### 2.4 Stereotaxic surgery

Male C57BL6J mice were acclimated for 7 days prior to the surgical procedure with ad libitum access to food and water. Animals were 2–2.5 months old and weighed 25–30 g at the beginning of all surgical procedures.

Presurgical procedures for stereotaxic surgery included anesthesia using a mixture of ketamine (Ketamil, 100 mg/mL; Troy Laboratories, Glendenning, Australia) and xylazine (Xylazil-20, 20 mg/mL; Troy Laboratories, Glendenning, Australia), diluted in normal saline at a ratio of 1:1:8 (ketamine:xylazine:saline). The mixture was administered intraperitoneally at doses of 100 mg/kg-body-weight for ketamine and 20 mg/kg-body-weight for xylazine.

Mice were placed on a pre-warm heating pad for 10 min post-anesthesia administration to avoid potential hypothermia. Upon loss of consciousness (confirmed by loss of reflexes), the head area was shaved and the mouse was kept for additional 2 min on the heating pad (as elevated heart rate was observed following the shaving procedure). Administration of artificial tear drops was conducted to avoid corneal desiccation as blink reflex is lost during anesthesia. Animals displaying breathing irregularity reaction to anesthesia (rate or depth of respiration) were kept on the heating pad for longer duration, if the symptoms persisted, the animal was excluded from the study.

During the surgical procedure, a midline incision was made at the top of the mouse’s skull to expose the skull surface and thus the stereotaxic landmarks, namely, bregma and lambda, using a surgical scalpel blade (No. 11). Striatal coordinates for injection were measured from the bregma as follows: +0.5 mm for the anterior–posterior side, −2.2 mm for medial-lateral, and −3.4 mm for dorso-ventral (Supplementary Figure 1). Human wild type (WT) α-syn PFF was inoculated into the striatum using a 10 μL Microliter Syringe (Model 701 RN, 26 s gauge; Hamilton, Timis County, Romania) connected to an automatic pump (Micro4^™^ MicroSyringe Pump Controller, WPI, Sarasota, FL, United States). The PFF, with a concentration of 1.44 mg/mL, was infused at a rate of 0.5 μL per minute, totaling 3.5 μL over a 7-min period, with an additional 1-min interval to ensure its proper diffusion.

Following the injection of PFF seed, the incised skin edges were closed using forceps and a tissue adhesive (3M Vetbond^™^/MC, Kowloon, Hong Kong). Phosphate-buffered saline (PBS) was injected in the same manner in the control mice group. Animals were kept on the heating pad following the surgical procedure until consciousness was regained. Subsequently, mice were individually housed in cages and continuously monitored throughout the study period.

Wound situation, temperature, body weight, and signs of stress were monitored. Signs of stress were usually halted 24 h following the stereotaxic surgery. Animals displaying overt signs of sickness were administered extra dose of analgesics to minimize post-operative pain, antibiotic ointment was applied onto the surgical wound. Pain medication was not routinely included in this study as its incorporation might have interfered with the inflammatory immune response investigated in this study.

### 2.5 Generation of human WT α-syn PFF seeds

Monomeric recombinant human α-syn was isolated and purified for the subsequent generation of fibrillar synuclein as mentioned earlier (Merghani et al., 2021). The expression vector pT7-7 wt-α-syn, a generous gift from Hilal Lashuel (Addgene plasmid #36046), was employed for the expression of the monomeric synuclein, and the construct was transformed into *Escherichia coli* BL-21 (DE-3). The harvested cells were resuspended in a non-denaturing lysis buffer (PBS containing 5 mM EDTA and 0.02% sodium azide), followed by homogenization with a glass homogenizer, sonication (10 min), boiling (10 min), and cooling on ice (30 min). Subsequently, the cell lysate underwent centrifugation (15k rpm for 20 min), and the supernatant was collected and dialyzed in the gel filtration buffer (10 mM Tris, pH 7.6, 50 mM NaCl, 1 mM EDTA, 1 mM PMSF). Lysate was filtered (using 0.22 μm filters) and concentrated (2–4 mL/L of cell culture) via a protein concentration column (MWCO 7K). The concentrated lysate was injected into a gel filtration column (Superdex 200), and fractions were collected and subjected to SDS gel separation for the protein of interest. BCA Assay was then conducted for selected fractions to determine their concentrations. To convert the purified monomeric synuclein into fibrils, α-syn (100 μM) was incubated at 37 °C for 7 days with constant shaking at 800 rpm in a Thermomixer (Eppendorf, Germany). Thioflavin-S assays were performed at various time intervals to monitor the fibril production process. The samples were subsequently centrifuged at 18k g for 10 min, and the insoluble fibrils were collected to prepare the PFF seeds. These fibrils were further fragmented on ice through ultrasonication using a Sonic ruptor 250 equipped with a fine tip (pulse duration: 5 s, 40 W, time: 5 min) (Karampetsou et al., 2017).

### 2.6 Brain collection for immunohistochemistry/immunofluorescence experiments

Animals were anesthetized with ketamine HCL—xylazine HCL mix and underwent perfusion with normal saline and PBS containing 4% paraformaldehyde (PFA). Following perfusion and brain extraction, the brains were placed in glass vials with 4% PFA and left overnight at 4 °C. Subsequently, the solution was replaced with a 10% sucrose solution (containing 0.1 M PB and 0.02% sodium azide) twice a day for three days. Any excess water remaining on the brain was absorbed and removed using paper towels, after which the brains were preserved at −80 °C for future use.

### 2.7 Cryo-sectioning of mouse brain

The mouse brain was sectioned into sequential slices of 40 μm thickness, extending from the rostral to the caudal end, using a cryostat (Leica CM1860 UV; Leica Biosystems, Wetzlar, Germany). These sections were utilized for evaluating various parameters, including the assessment of phosphorylated synuclein and the intensity of DA neuron terminals in the STM, detection of α-syn (both phosphorylated and proteinase K resistant species), neuroinflammatory markers ionized calcium binding adaptor molecule 1 (IBA-1) and glial fibrillary acidic protein (GFAP) in the SNC, and the count of tyrosine hydroxylase (TH) positive DA neurons.

### 2.8 Immunohistochemistry

#### 2.8.1 3,3′-Diaminobenzidine staining

Sections underwent three washes (10 min each) with 0.01 M PBS, pH 7.4, followed by blocking in 10% normal goat serum in PBS containing 0.3% Triton-X 100 (PBST) at room temperature for 1 h. Subsequently, the sections were washed with 0.01 M PBS and incubated with the specific primary antibody (Table 1) for 48 h at 4 °C. Secondary staining was carried out with biotin streptavidin antibody to enhance sensitivity. After PBS wash, the brain sections were exposed to horseradish peroxidase conjugated streptavidin (Sigma, Streptavidin-HRP Conjugate, 1 in 200) for an hour. 3,3′-Diaminobenzidine (DAB) reaction enabled visualization of the immunoreaction. Finally, the sections underwent defatting, after which Epredia^™^ Shandon^™^ Synthetic Mountant medium (Fisher Scientific, Hampton, New Hampshire, United States) was used.

**Table 1:** List of antibodies.

Antibody	Catalog No. (host)	Source	Dilution	Assay
Primary				
Tyrosine hydroxylase	Immuno star 22941 (mouse)	ImmunoStar, Hudson, Wisonsin, United States	1:1,000	IF/DAB
Tyrosine hydroxylase	AB152 (rabbit)	Merck, Burlington, MA, United States	1:1,000	IF
DAT	TEMECULA MAB369 (rat)	Merck, Burlington, MA, United States	1:1,000	DAB
Phospho-synuclein (pS129-α-syn)	ab59264 (rabbit)	Abcam, Waltham, MA, United States	1:1,000	IF
Filamentous α-synuclein	ab209538 (rabbit)	Abcam, Waltham, MA, United States	1:2,500	IF
GFAP	G3893 sigma (mouse)	MilliporeSigma, Burlington, MA, United States	1:1,000	IF
IBA-1	Fujifilm Wako; 019-19741 (rabbit)	Fujifilm Wako, Osaka, Japan	1:1,000	IF
Secondary				
Biotin-sp-conjugated	Jackson Immuno Research 712-065-153 (donkey anti-rat)	Jackson ImmunoResearch Laboratories, West Grove, PA, United States	1:1,000	DAB
Biotin-sp-conjugated	Jackson Immuno Research 715-065-150 (donkey anti-mouse)	Jackson ImmunoResearch Laboratories, West Grove, PA, United States	1:1,000	DAB
Alexa Fluor 594	Invitrogen; A11032 (goat anti-mouse)	Thermo Fisher Scientific Pierce Biotechnology, Rockford, IL, United States	1:1,000	IF
Alexa Fluor 488	Invitrogen; A11034 (goat anti-rabbit)	Thermo Fisher Scientific Pierce Biotechnology	1:1,000	IF
Alexa Fluor 594	Invitrogen; A-11012 (goat anti-rabbit)	Thermo Fisher Scientific Pierce Biotechnology, Rockford, IL, United States	1:1,000	IF
Alexa Fluor 488	Invitrogen; A-11001 (goat anti-mouse)	Thermo Fisher Scientific Pierce Biotechnology	1:1,000	IF

##### 2.8.1.1 Tyrosine hydroxylase immunostaining of SNC neurons

Every sixth coronal section, spanning from the rostral to the caudal end and covering the area of −2.18 to −3.80 mm of bregma, were collected from each brain sample for immunostaining experiments (Supplementary Figure 2). The quantification of DA neurons, identified by positive staining with the TH antibody, was conducted using an unbiased stereological approach. The stereo-investigator system (version 2020) employed an optical fractionator to estimate the number of DA neurons in the SNC area. Following the instructions provided by the manufacturer, seven coronal sections from the SNC area of each animal were systematically counted in serial order, as per mouse brain atlas (Paxinos and Franklin, 2013). The *z*-axis microcreator facilitated the measurement of section thickness during counting. A counting contour was delineated in the region of interest at 5× magnification, and the count was obtained using 63× magnification. The results are presented based on the number of TH-positive neurons in the SNC.

##### 2.8.1.2 Dopamine transporter staining of striatum

The visualization of the avidin-biotin complex peroxidase reaction with DAB was conducted following previously optimized protocols (Merghani et al., 2021). Image J software (NIH, Bethesda, MD, United States) (version: Image J 1.54d) was utilized to analyze the results, specifically assessing the decrease of striatal fibers density in the striatum. The staining dopamine fiber was performed using a polyclonal rat antibody for DAT (MAB369, 1:1,000).

### 2.9 Immunofluorescence and confocal microscopy

Brain sections underwent washing with 0.01 M PBS, pH 7.4, and blocking with 10% normal goat serum in PBST at room temperature for one hour after which the sections were washed once with 0.01 M PBS and stained with primary antibodies (Table 1). Two days after incubation with the primary antibodies, sections were washed with 0.01 M PBS (three times) and incubated at 4 °C overnight with their corresponding secondary antibodies. Following this, sections were washed with PBS and mounted on a slide using fluoroshield mounting medium (Sigma), and images were acquired using a confocal microscope (Nikon EZC1). As a confirmation of the success of the surgery, striatal tissue sections were stained with rabbit polyclonal antibody against α-syn phosphorylated at serine129 (pS129-α-syn), a typically used marker that is found to aggregate and form upon PFF administration (Gómez-Benito et al., 2020). For investigating spreading and accumulation of α-syn, SNC sections were co-stained with mouse primary monoclonal antibody against TH and rabbit polyclonal antibody against pS129-α-syn or anti-α-syn filament-specific antibody.

To assess filamentous α-syn species, soluble α-syn was digested by pretreating the brain sections with proteinase K (PK) at 5 μg/mL for 30 min at 25 °C. After PK digestion, the sections were washed three times with PBST (5 min each), blocked for 1 h with 10% normal goat serum, and co-stained with primary antibodies against filamentous α-syn and TH, following the previously described protocol.

For neuroinflammation analysis, SNC sections were stained with mouse anti-GFAP antibody and rabbit primary polyclonal anti-IBA1 antibody. Sections stained with IBA-1 underwent prior treatment with an antigen-retrieving agent (Proteinase K, 5 μg/mL, for 30 min at 25 °C).

### 2.10 Assessment of α-syn colocalization with TH positive neurons; and activated microglia and astrocytes

#### 2.10.1 α-synuclein spreading

The analysis of pS129-α-syn and α-synuclein aggregate expression (representing the phosphorylated and filamentous forms, respectively) within dopaminergic neurons was conducted using a sample size of *n* = 3–4 per group. Brain sections encompassing the substantia nigra pars compacta (SNc) were stained for each animal, following a collection method similar to that used for DAB staining of TH neurons, as described above. For analysis, three sections per animal (corresponding to bregma levels −2.92 mm, −3.08 mm, and −3.16 mm) were selected, as these regions exhibited the greatest overlap of synuclein with TH-positive neurons. From each section, up to three fields of view were acquired, maintaining a consistent field area across all samples. The ratio of TH-positive neurons co-localizing with either pS129 or filamentous α-syn aggregates to the total number of TH-positive neurons in each field was calculated and expressed as a percentage. The mean percentage of colocalization per group was then determined, and statistical analyses were performed and presented graphically.

#### 2.10.2 Neuroinflammation

Five to six rectangular regions of interest (ROI) of the same area were created over the SNC region, in which activated astrocytes and microglia (depicted by star-like projections) are counted using ImageJ software. The information is displayed as the average number of activated astrocytes or microglia counted in each field or ROI. Leica DM4000 B LED Microscope (Leica Microsystems, Wetzlar, Germany) was used to capture images.

### 2.11 Statistical analysis

One-way ANOVA followed by Bonferroni’s Multiple comparison test for analysis of more than two groups. Unpaired (independent) *t*-test was used for comparison between two groups only. Statistical analysis was conducted using GraphPad Prism 5 software (InStat software, La Jolla, CA, United States). All data is shown as mean ± SEM (*n* = 3–4) with significance denoted as follows: ns, not significant, ^*^p < 0.05, ^**^p < 0.01, ^***^p < 0.001, ^****^p < 0.0001. Three animals per group were included in the analysis.

## 3 Results

### 3.1 Injection of human WT α-syn PFF in striatum induces endogenous phospho-synuclein expression

**Figure 2:**
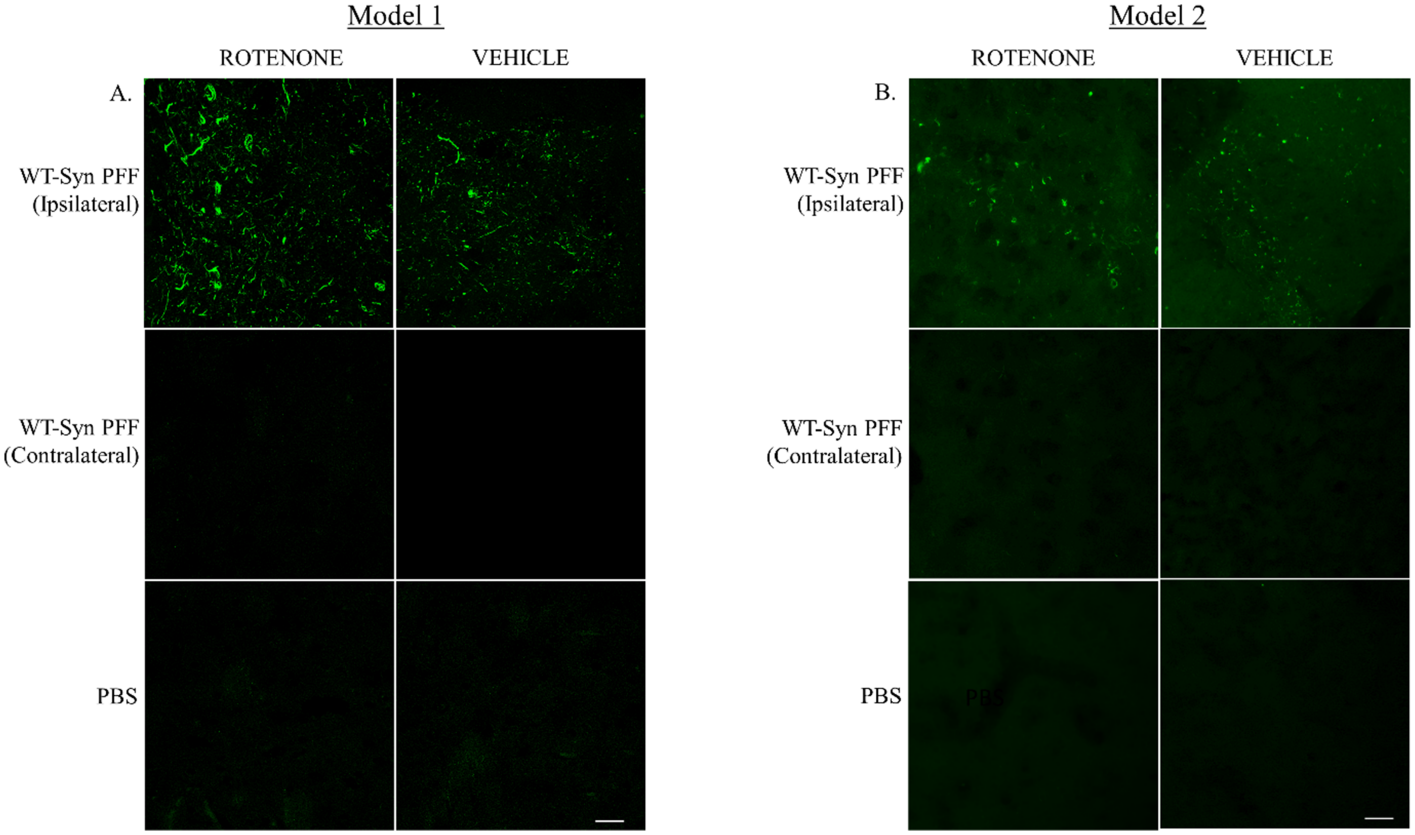
Intra-striatal injection of human WT α-syn PFF in the mouse brain. Human α-syn PFF seeds were injected into the striatum of C57BL6 black mice via stereotaxic surgery. **(A)** Animals were administered with rotenone (2.5 mg/kg-body-weight) or vehicle through IP route on once daily for 4 weeks (Model 1). Twenty-four hours after the last rotenone injection, brains were extracted for IHC analysis. Coronal sections of the striatal tissue were immuno-stained with pS129-α-syn. **(B)** In Model 2, additional 3-week gap was introduced between the stereotaxic surgery and rotenone administration. Representative images depict signals for pS129-α-syn at the injected site (striatum) in PFF injected mice. Scale bar: 25 μm.

The present study assessed the effect of rotenone administration in a PFF (intra-striatal injection) model of PD utilizing black mice (C57BL6J). Two different durations of experimental models employing the same dose of rotenone were explored. Model system 1 is a 4-week plan (Figure 1A) where rotenone is delivered one day after stereotaxic surgery of PFF into the mouse STM, and Model system 2 is a 7-week plan in which the PFF seed is allowed to spread for 3 weeks, after which rotenone is administered for 4 weeks in the same manner (Figure 1B). The present study examines if rotenone exerts any role in the accumulation and spreading of α-syn. First, we verified successful conduction of intra-striatal stereotaxic surgery. Clear signals of pS129-α-syn were observed in the STM of both model systems (Figures 2A,B) in PFF injected mice that received either vehicle or rotenone. No signal was observed in the contralateral side of the striatal sections and (internal control) in animals injected with PBS (external control). This confirmed PFF’s role as a seeding agent for induction and/or expression of endogenous α-syn.

### 3.2 Rotenone administration enhances spreading of α-syn in the SNC of PFF injected mice

After confirming the induction of endogenous α-synuclein following PFF injection in the STM, this study investigated the effect of rotenone administration on PFF-α-syn spreading via retrograde transport from the nerve terminals in the STM to the cell bodies in the SNC, and the resulting α-syn aggregation. SNC sections were double-labeled using immunofluorescence (IF) with anti-TH (a marker for DA neurons) and anti-pS129-α-syn.

**Figure 3:**
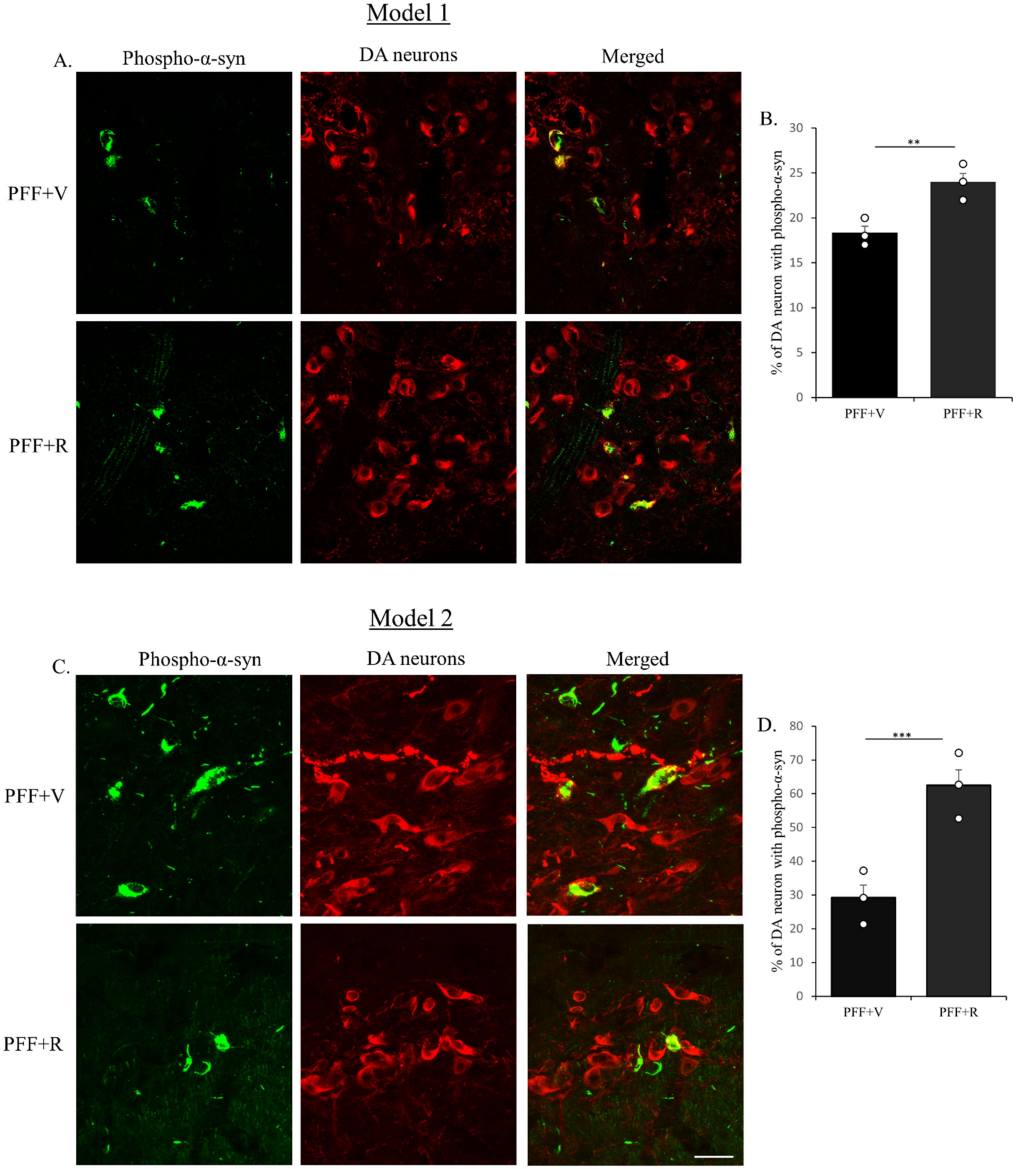
Coronal sections of mouse SNC were co-labeled with anti-pS129-α-syn to detect endogenous phospho-α-syn (green) and anti-TH antibodies to detect dopaminergic (DA) neurons (red). Co-staining for Model 1 **(A)** and Model 2 **(C)** shown. PFF + R group displayed higher accumulation of pS129-α-syn in both models **(B,D)**. Taken together, rotenone administration enhanced the spreading of endogenous phospho-α-syn in human WT α-syn PFF injected mice in the SNC area. Scale bar: 50 μm. Data represents mean ± SEM (*n* = 3). ^***^p < 0.001 and ^**^p < 0.01.

An enhanced accumulation of pS129-α-syn α-syn within TH-positive neurons was observed in PFF-injected mice treated with intraperitoneal rotenone in both experimental models (Figures 3A,C). The total number of TH-positive neurons in the SNC co-localizing with pS129-α-syn was quantified. A significant increase in pS129-α-syn accumulation and its co-localization with TH-positive neurons was evident in the PFF + rotenone (PFF + R) group compared to controls in both models (Figures 3B,D). Notably, the enhancement in α-syn spreading in the PFF + R group was nearly fivefold greater in Model system 2 compared to Model system 1, with a correspondingly higher level of statistical significance.

### 3.3 Rotenone enhances the proteinase-K resistant (nuclear) filament specific α-syn in α-syn injected mice

**Figure 4:**
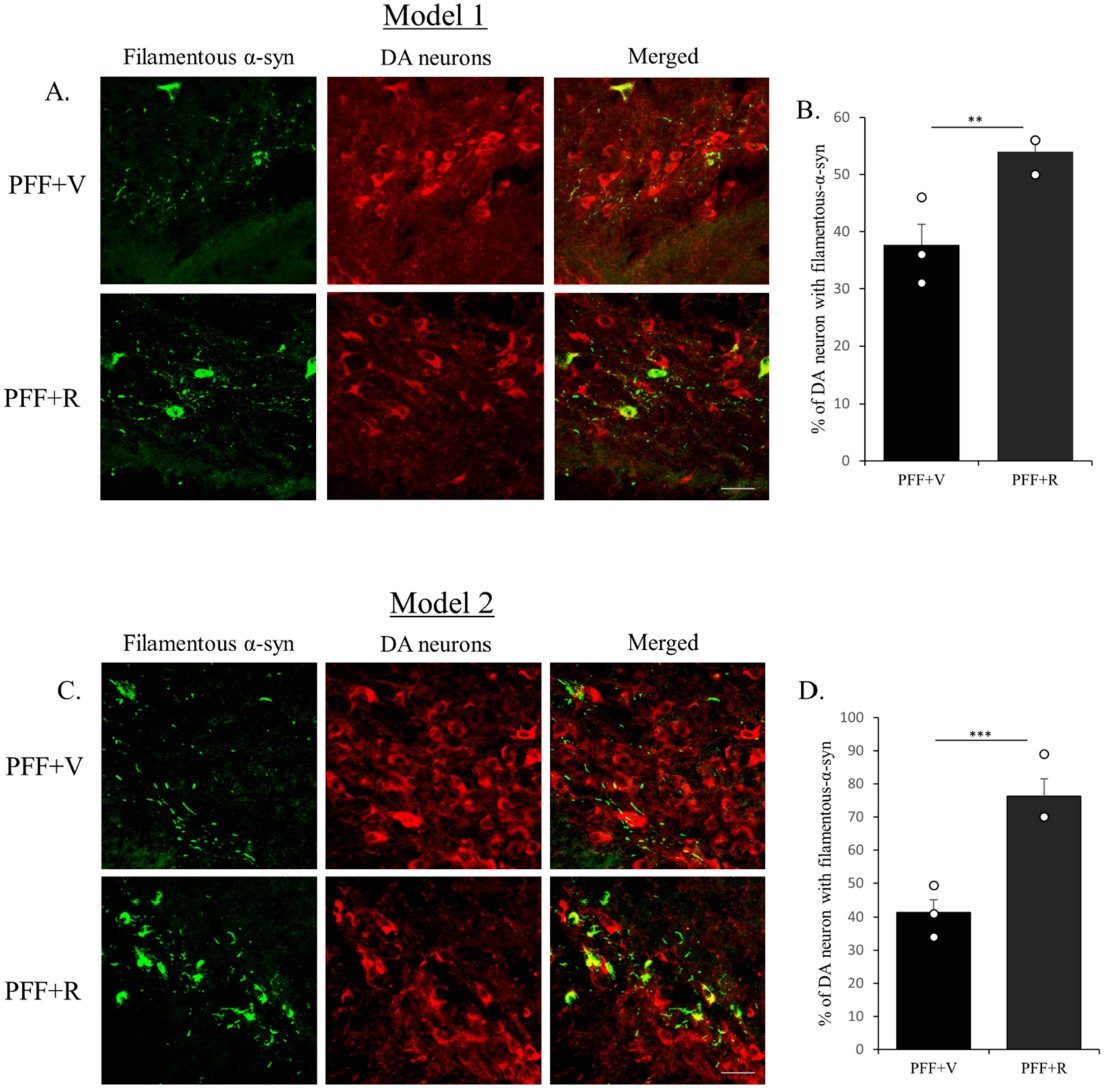
Rotenone administration enhanced the accumulation of filamentous α-syn in human WT α-syn PFF injected mice. The coronal section of the SNC area was stained with aggregate specific α-syn antibody to detect filamentous α-syn (green) and dopaminergic (DA) neurons (red) (Model 1: **A,B** and Model 2: **C,D**). Scale bar: 50 μm. The number of TH positive neurons that co-localize with filament specific α-syn was counted and presented as a percentage in the bar graph. Data represents mean ± SEM (*n* = 3). ^***^p < 0.001 and ^**^p < 0.01.

**Figure 5:**
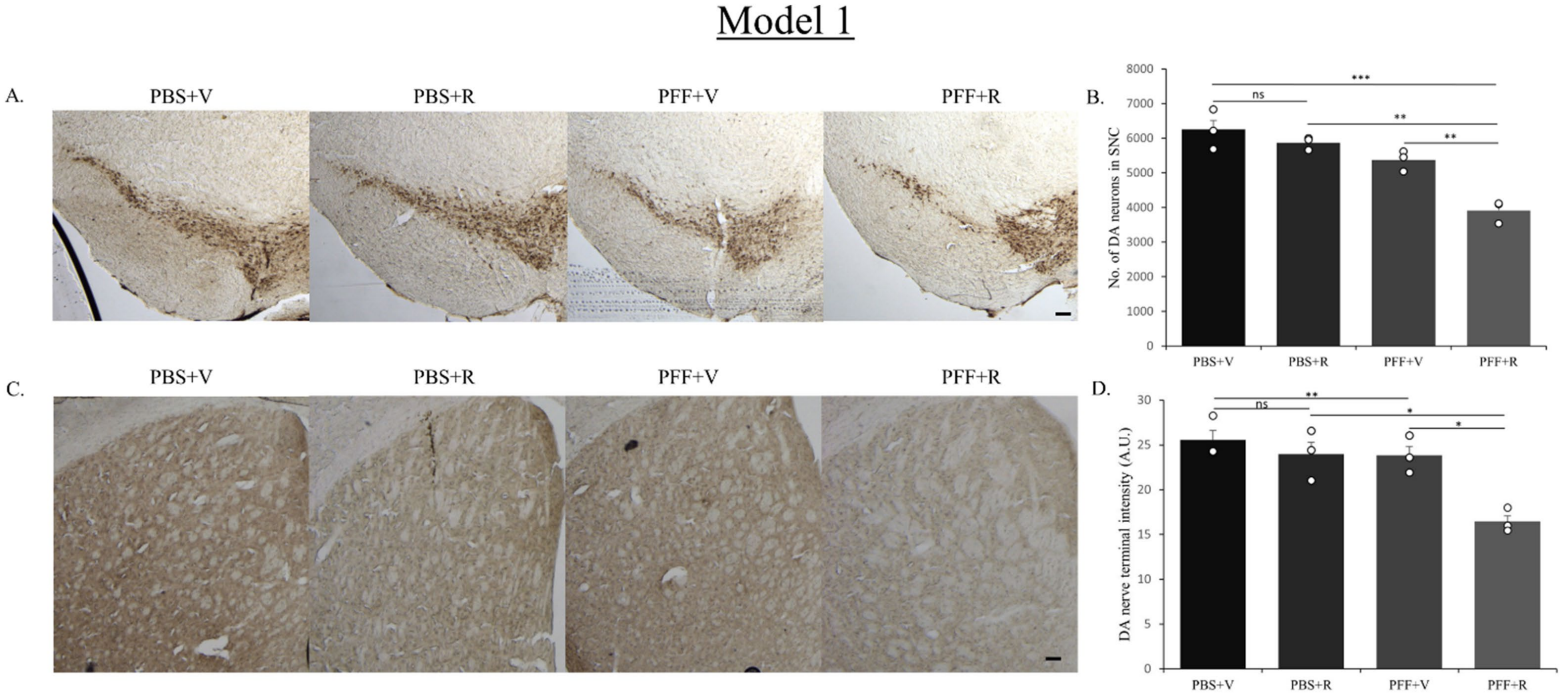
Representative image showing TH neurons in the SNC region **(A)** and DAT staining of STM region **(C)**. Representative images show a decrease in TH neuronal number in the SNC **(B)** and DAT intensity in the STM **(D)** of the PFF + R mice as compared to the PFF + V mice. Scale bar: 100 μm. Data represents mean ± SEM (*n* = 3). ^***^p < 0.001, ^**^p < 0.01, ^*^p < 0.05, and ns, not significant.

Previous studies have indicated that detectable α-syn molecules after PK digestion signify the presence of aggregated synuclein (Forloni, 1996; Neumann et al., 2004; Tanji et al., 2010; Yoshinaga et al., 2020). This is essentially an indirect method to determine the presence of Lewy body-like synuclein inclusions due to their resistance to PK digestion. It is important to note that PK digests the monomeric physiological synuclein but is unable to digest the fibrillar pathogenic forms of synuclein (Forloni, 1996). In our investigation, we observed an accumulation of Lewy body-like α-syn inclusions in the TH neurons of the animals inoculated with α-syn PFF. The experimental group PFF + R exhibited a more substantial α-syn accumulation (Figures 4A,C). Consistent with the previous observation, the percentage difference in filamentous α-syn accumulation in PFF + R mice compared to PFF + V mice was almost 2-fold higher in Model system 2 (~35%) than in Model system 1 (~16%) (Figures 5B,D).

**Figure 6:**
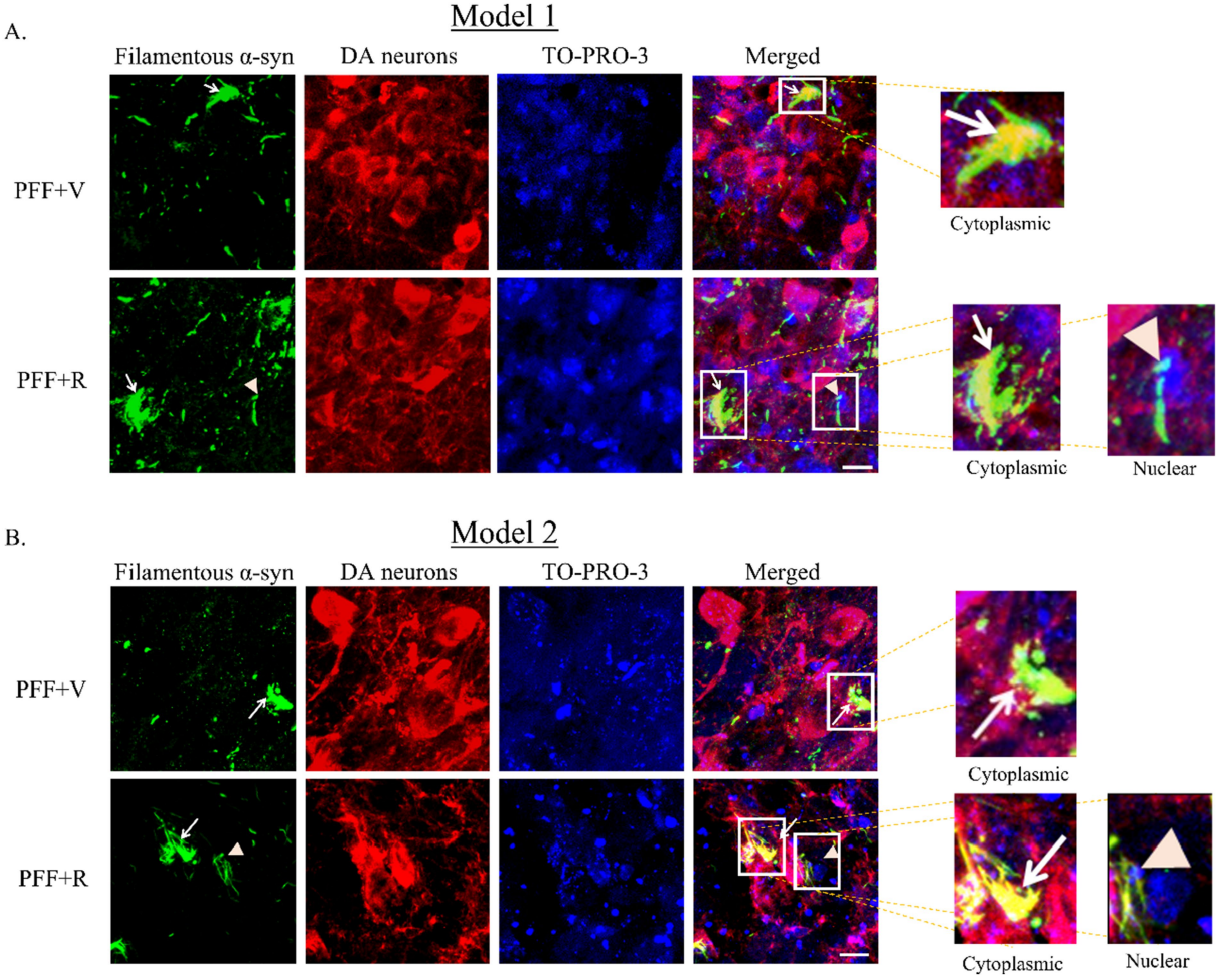
Coronal section of the SNC area stained with an aggregate specific α-syn antibody to detect filamentous α-syn (green), DA neurons (red) and TO-PRO-3 nuclear marker (blue) (Model 1: **A**, Model 2: **B**). Representative images showing accumulation of filamentous specific α-syn in vehicle and rotenone treated groups. White arrow denotes cytoplasmic inclusion and orange arrowhead denotes nuclear inclusion of synuclein. Scale bar: 25 μm.

During the analysis of the filamentous α-syn in TH-positive neurons, we noted a tendency for nuclear localization in PFF + R group of mice (of both models), which was not the case for PFF + V group (Figures 6A,B). Further investigation of nuclear accumulation of filamentous α-syn should be conducted before drawing definitive relation between rotenone and nucleo-cytoplasmic transport of α-syn aggregates.

### 3.4 Rotenone enhances nigrostriatal neurodegeneration in mice injected with PFF

Next, we investigated the effect of rotenone on the nigrostriatal-tract of the PFF injected mice in both the models. We assessed whether it correlates with the increase in spreading and aggregation of α-syn. An unbiased stereo-investigator system was employed to quantify the total number of DA neurons in the SNC.

**Figure 7:**
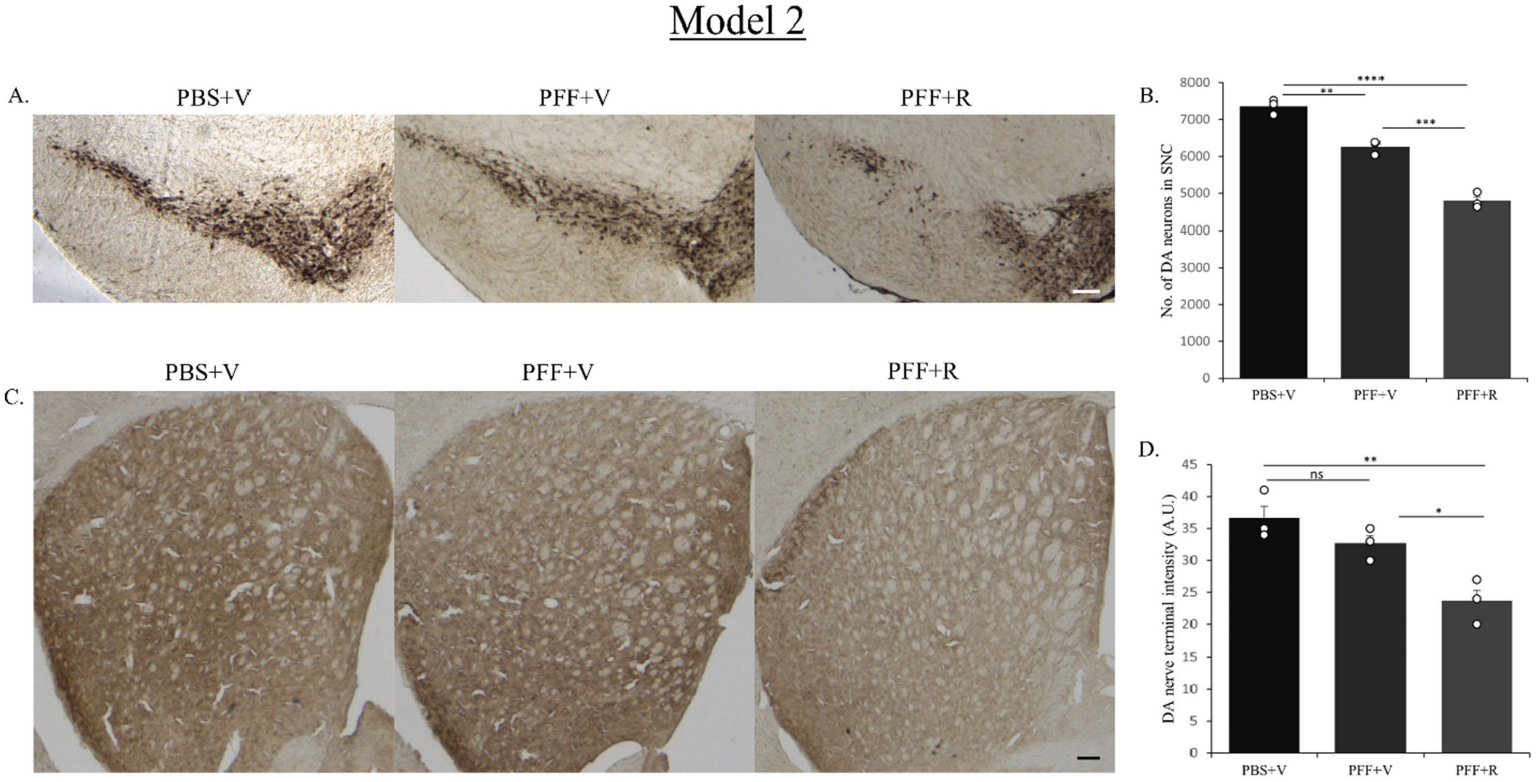
Representative image showing TH neurons in the SNC region **(A)** and DAT staining of STM region **(C)**. Representative images show a decrease in TH neuronal number in the SNC **(B)** and DAT intensity in the STM **(D)** of the PFF + R mice as compared to the PFF + V mice. In Model system 2 also, approximately 20% reduction in DA neuronal number in SNC was observed upon administration of rotenone to PFF injected mice (again considering the PBS + V group as “100”). Scale bar: 100 μm. Data represents mean ± SEM (*n* = 3). ^****^p < 0.0001, ^***^p < 0.001, ^**^p < 0.01, ^*^p < 0.05, and ns, not significant.

The PBS-only group of mice in both model systems exhibited the highest number of DA neurons in the SNC (Figures 5A, 7A). In Model system 1, we observed that the rotenone-only group (which received intra-striatal PBS injection and intraperitoneal doses of rotenone) did not induce neurotoxicity on its own (Figure 5B). Hence, for Model system 2 we proceeded to assess three experimental groups: namely, PFF + R, PFF + V, and PBS + V (Figure 7). On comparing the PBS + V mice with the PFF + V group of mice, we observed that the presence of PFF did indeed result in a slight reduction in DA neuronal number. This neurodegeneration was further intensified upon the administration of rotenone to the PFF-injected mice in both of our model systems (Figures 5B, 7B). Our findings from nigral DA neuronal staining highlight the role of rotenone in enhancing synuclein-induced toxicity and neurodegeneration. Given that the nerve endings of DA neurons in the SNC region are present in the striatum, the observed neuronal reduction should correlate with the intensity of nerve terminals in the STM (Gómez-Benito et al., 2020). To investigate this, DA nerve terminals in the STM were stained with antibodies against the dopamine transporter (DAT). In line with the observed neuronal loss, we found that the PBS + V group in both models had the highest intensity for DAT (Figures 5C, 7C). Once again, this intensity was comparable to the PBS + R group of Model system 1, reaffirming that rotenone by itself does not induce any reduction in DAT density or nigrostriatal tract damage in our experimental setup. The administration of PFF led to a reduction in dopamine terminal intensity in the striatum sections of both model systems (Figures 5D, 7D). This intensity was further reduced upon rotenone administration. Thus, rotenone administration augmented nigrostriatal tract damage in PFF-injected mice, as evidenced by the reduction in DA neuronal number in the SNC and DA nerve terminal intensity in the STM.

### 3.5 Rotenone treatment enhanced the activation of astrocytes and microglia in the mice inoculated with PFF

Another key feature associated with PD pathology is neuroinflammation. The activation of microglia and astrocytes is an important indicator of neuroinflammation linked to PD (Kaur et al., 2017; Lull and Block, 2010; Wang et al., 2015). Therefore, in our model, we assessed neuroinflammation, by staining the SNC sections with IBA-1 and GFAP antibodies, as markers of microglia and astrocytes, respectively. Microglial activations have been associated with increased oxidative stress, and deficits in motor and cognitive functions in PD rodent model systems. In a recent study, rotenone was reported to raise the levels of GFAP and IBA-1 in an albino rat model system for PD (Azimullah et al., 2023). These results demonstrate how rotenone contributes to the development of observed neuropathology by causing neuroinflammation in various PD model systems. Thus, it becomes essential to evaluate and validate the impact of rotenone in a conjunctive manner with intra-striatal PFF to assess the immune response in a PD mouse model system.

**Figure 8:**
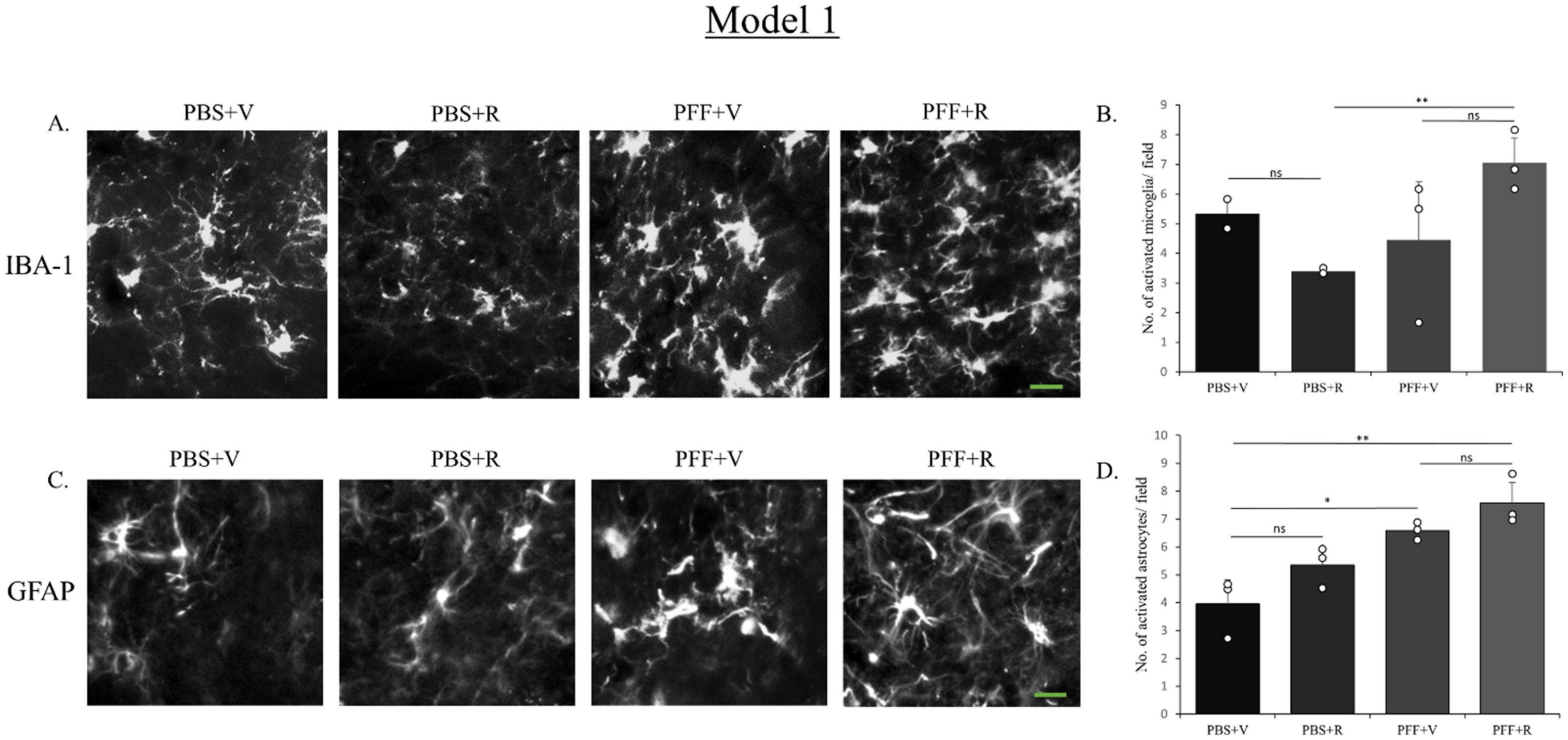
Microglia and astrocyte activation after rotenone administration in the animal pre-inoculated with PFF (Model 1). SNC of the coronal sections were stained with antibody IBA-1 **(A)** and GFAP **(D)** to detect activated microglia and astrocytes, respectively. Images (originally green for IBA-1, and red for GFAP) have been converted to grayscale. PFF + R mice showed general increase in the number of activated microglia as well as astrocytes, however this increase was not statistically significant. Scale bar: 15 μm. The number of activated microglia **(B)** and astrocytes **(D)** at the SNC per field was counted and presented **(B,D)**. Data represents mean ± SEM (*n* = 3–4). ^**^p < 0.01, ^*^p < 0.05, and ns, not significant.

In the current study, mice receiving both intra-striatal PFF and Rotenone exhibited higher activation of microglia and astrocytes in both model systems. While in model 1, the microglial and astrocytic activation between the groups receiving intra-striatal PFF injection (in rotenone and vehicle treated groups, respectively) was not statistically significant (Figures 8B,D), the IP administration of rotenone alone (without prior PFF treatment) displayed a significantly lower rate of microglial activation. This is indicative of the synergistic neurotoxic effect that is probed by rotenone treatment in presence of PFF intra-striatal inoculation (Figures 8A,C).

**Figure 9:**
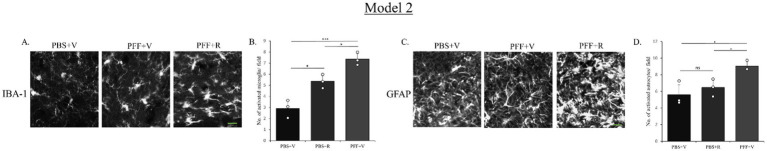
Microglia and astrocyte activation after rotenone administration 3 weeks after intra-striatal PFF injection (Model 2). SNC of the coronal sections were stained with antibody IBA-1 **(A)** and GFAP **(C)** to detect activated microglia and astrocytes, respectively. Images (originally green for IBA-1 and GFAP) have been converted to grayscale. PFF + R mice showed statistically significant increase in the number of activated microglia as well as astrocyte. Scale bar: 15 μm. The number of activated microglia **(B)** and astrocytes **(D)** at the SNC per field was counted and presented **(B,D)**. Data represents mean ± SEM (*n* = 3–4). ^*^p < 0.05 and ns, not significant.

Model 1 was modified by a 3-week gap kept between the PFF intra-striatal injection and the initiation of rotenone IP administration (Model 2). This modification aggravated the neuroinflammatory response mediated by rotenone in presence of PFF inoculation (Figures 9A–D). This change could be attributed to the enhanced α-syn spreading (within the 3-week gap) prior to the induction of oxidative stress and neurotoxicity conferred by rotenone treatment.

## 4 Discussion

Approximately two decades ago, chronic exposure to systemic rotenone in rats demonstrated replication of key features of PD (Betarbet et al., 2000). This study provided initial histopathological evidence of Lewy body-like inclusions accumulating in nigral DA neurons *in vivo* using rat model. Rotenone-induced neurotoxicity model of rat had been utilized in many neuroprotective studies (Al-Abbasi, 2022; Magdy et al., 2022; Sarkaki et al., 2016). Very few studies explored the potential of rotenone in induction of PD-like pathology in mice which are more commonly utilized in neuropathological studies (Sanfeliu et al., 2024).

Nevertheless, the rotenone-induction of PD model holds significant potential for further improvement. The adjunction of rotenone administration in this study with initial intra-striatal α-syn PFF is, to the best of our knowledge, the first study to investigate the synergistic neurotoxicity between induced internal α-syn aggregation (via intra-striatal PFF injection) and rotenone IP administration in a mouse model. Both models investigated in this study were planned in an attempt to develop a PD mouse model system which could closely mimic PD’s synucleinopathy.

In this study two distinct model systems were employed to investigate the impact of rotenone on enhancing α-syn-induced PD pathology in C57BL6J black mice. The concurrent initiation of the neurotoxicity via induction of oxidative stress conferred by rotenone along with the presence of intra-striatal PFF in the first model system, aims to emulate PD where synuclein pathology is not initially present, exploring how the combination of rotenone exposure with fibrillar structures influences α-syn aggregation pattern, spreading and neuroinflammatory response. In the second model system, rotenone was administered three weeks after the PFF delivery, evaluating its effect on enhancing synuclein pathology already present in the mouse. This model is closer to the case of sporadic PD, examining how exposure to environmental toxicants affects the further progression of the disease in individuals who already exhibiting susceptibility to synuclein pathology. Both models aim to demonstrate the synergistic role of rotenone in enhancing PD-like features induced by intra-striatal PFF in a mouse model system.

In this study, results indicated an increased spreading and accumulation of both phosphorylated, and PK resistant, filamentous α-syn in the PFF + R group of both models. Additionally, the pathogenic PK resistant α-syn species displayed a tendency to colocalize with the nucleus of the nigral DA neurons in the PFF + R group. Nuclear species of α-syn have been implicated in conferring neurotoxicity via inhibition of histone acetylation (Du et al., 2024; Kontopoulos et al., 2006). Taken together, this evidence suggests that nuclear α-syn in the PFF + R group may play a neurotoxic role. However, further quantitative experiments are necessary to clarify the role of nuclear localization pattern observed in the models proposed in this study.

After observing an increase in the spreading and aggregation of α-syn, next, the investigation of association between accumulated synuclein and DA neurons viability in the nigrostriatal pathway was conducted. It was observed that there was indeed a reduction in the number of nigral DA neurons and its terminals residing in the STM in mice that received both PFF and rotenone, thereby indicating damage to the nigrostriatal tract in both models.

Another factor that plays a pivotal role in the progression of PD, is neuroinflammation, evidenced by activated astrocytes and microglia (Kaur et al., 2017; Lull and Block, 2010; Wang et al., 2015). Therefore, in addition to investigating the two key features of PD (synuclein spreading and neurodegeneration) assessment of neuroinflammation was conducted in this study. While Model 1 displayed a general trend of microglia and astrocyte activation, via the detection of IBA-1 and GFAP in the SNC, respectively, acquired results from Model 1 were not statistically significant. In Model 2, however, the 3-week gap provided prior to the induction of rotenone treatment seemed to confer higher capacity of α-syn propagation and aggregation all of which could have aided in a higher neuroinflammatory response.

Collectively, IHC analysis revealed that the LB-like pathology was more significant in Model system 2 hinting towards the value of an incubation period between the initial PFF inoculation (striatal) and the initiation of rotenone treatment as a preferable PD animal model, nevertheless, a systematic comparison of the two model systems is necessary for confirmation.

Limitation of the present study includes conduction of behavioral tests, the addition of which would further support the characterization of this PD model. Further investigation of α-syn translocation to DA neurons’ nuclei upon rotenone exposure in PFF-injected mice will provide valuable insights into the mechanisms by which rotenone is able to exert its neurotoxicity on the transcriptional level.

## 5 Conclusion

Taken together, the study’s findings reveal that the IP administration of 2.5 mg/kg-body-weight rotenone in mice injected with intra-striatal PFF successfully enhances α-syn spreading, neurotoxicity, and neuroinflammation in the SNC area. This mouse model aims to emulate the etiology of sporadic PD and facilitate improved understanding of the synergistic role played by environmental and genetic influences. Such a model system holds promise for identifying therapeutic targets responsive to α-syn pathology, potentially contributing to the advancement of effective treatment strategies for PD.

## Data Availability

The raw data supporting the conclusions of this article will be made available by the authors, without undue reservation.
