# Supplementary material for: Rotenone accelerates endogenous α-synuclein spreading and enhances neurodegeneration in an intra-striatal α-synuclein preformed fibril injected mouse model of Parkinson’s disease
Source: Front Cell Neurosci. 2025 Oct 3;19:1624593. doi: 10.3389/fncel.2025.1624593 (PMC12531225; doi:10.3389/fncel.2025.1624593)
Supplement: Supplementary file 1 [file Data_Sheet_1.docx]

**Supplementary Figures
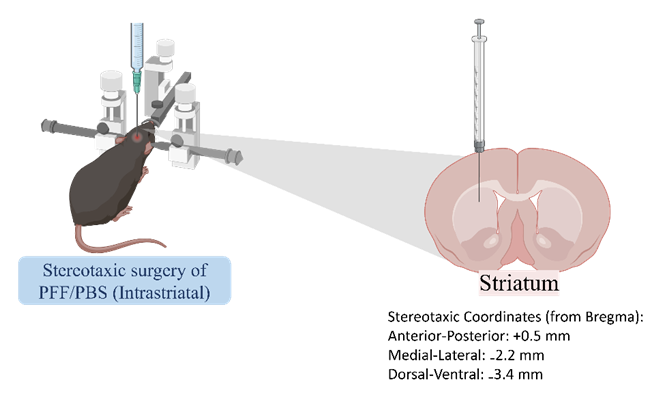
**

Supplementary Figure 1. Intra-striatal injection of PFF/ PBS. Figure illustrated using BioRender.


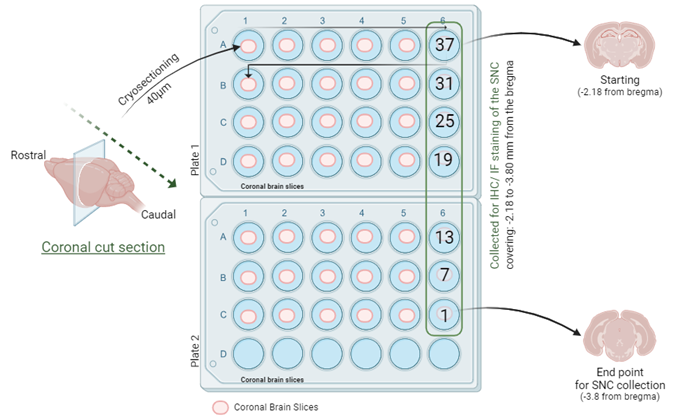


Supplementary Figure 2. Mouse brain coronal section collection for IHC & IF staining (each well contains only one section). Diagrammatic representation shows collection of SNC region. Illustrated using Biorender.
